# Evaluation of the effect of oral ritodrine on implantation rate in in-vitro fertilization-embryo transfer cycles

**Published:** 2011

**Authors:** Soghra Rabiee, Marziyyeh Farimani, Maryam Ahmadi

**Affiliations:** Department of Obstetrics and Gynecology, School of Medicine, Hamadan University of Medical Sciences, Hamadan, Iran.

**Keywords:** *Ritodrine*, *IVF*-*ET*, *Implantation rate*

## Abstract

**Background:**

Pregnancy rate with IVF cycle is almost 22%. Many investigations perform to increase this rate in IVF. Various factors affect the result of IVF cycles. One of these factors could be uterine contractions that expel transferred embryo. Ritodrine is a beta mimetic agent that can block and decrease uterine contractions.

**Objective:**

The objective of this study was to determine ritodrine effectiveness for increasing the implantation rate in IVF cycles, and its probable mechanisms in decreasing uterine contractions as well.

**Materials and Methods:**

A total of 100 patients of IVF-ET cycles were divided randomly in two groups in a university hospital, Hamadan, Iran. The case group were prescribed ritodrine 10 mg / bid orally after oocyte retrieval until 10 days. The control group didn’t received ridotrine.

**Results:**

In ritodrine group 14% of patients and in control group 16% had positive β-hCG test (p-value>0.5).

**Conclusion:**

Ritodrine did not improve the implantation rate in IVF-ET cycles.

## Introduction

Infertility is a common problem affecting up to 10% of married couples (1). A systematic evaluation of aetiologic factors forms the basis for choice of treatment and future fertility. On the global perspective, assisted reproductive technologies (ART) have become internationally recognized treatment option for some infertile couples. At present time, infertility treatments by ART is progressing and improving considerably (2). These treatments have improved the success rate of IVF-ET up to 22% (2). However, infertile couples spend much money and time especially in ART cycles and they expect more success rate. Presently , the maturation of follicles with gonadotropin, oocyte retrieval, in vitro fertilization and embryo development are satisfactory, but the main problem is why sometimes the embryo doesn't implant in the uterus successfully (3)? Different factors such as type of catheter for embryo transfer, using tenaculum for grasping the cervix, removing the obvious or excess cervical mucus before transfer, volume of transfer media, and embryo transfer under trans abdominal ultrasound guidance avoiding the uterine contraction at the time of embryo transfer, studied previously (4, 5). 

Ritodrine, as a beta- agonist, may improve the implantation rate in IVF cycles with decreasing uterine contraction and consequently decreasing extraction of embryos which transferred to the uterus (6). 

However, the results of studies are controversial in relation to controlling uterine contractions and its effect on implantation rate (7, 8). Some other chemicals such as hyoscine bromide, piroxicom and oxytocin antagonists has investigated and showed promising results (9-11).

The aim of this study was determination of ritodrine effect on uterine contraction and pregnancy rate in IVF-ET patient.

## Materials and methods

In a randomized controlled clinical trial, one hundred women aged between 25 through 35 years with a history of >5 year infertility enrolled to study. Inclusion criteria were as follow: having a morphologically normal uterus, infertility from tubal, male, endometriosis or unexplained factor. Excluding criteria were lack of any systemic known diseases in both groups. These patients were candidate for IVF-ET and were randomly allocated into treatment (50 patients) and control (50 patients) groups. Two groups had not significantly differences in view of mean age, causes of infertility and infertility duration. All the patients were stimulated with a GnRH agonist and exogenous gonadotropins with long protocol. On the day after oocyte retrieval, all patients in the case group received oral ritodrine 10 mg/ bid for ten days. Other medications in both groups were similar including ASA 80 mg/day, progesterone 100mg IM, erythromycin 400 mg/qid, and heparin 5000 u/bid. The luteal phase was supported by progesterone injection. The successfulness criterion was positive β-hCG after 2 weeks. At least three good quality embryos transferred for each patient and, the results of two groups (rate of positive β-hCG) compared and were analyzed with chi square test.

This study was done in a public center for infertility treatment and was approved in the Research Ethics Committee, Chancellor for Research and Technology, Hamadan University of Medical Sciences. 

**Statistical analysis**

The pregnancy rate between two groups (rate of positive β-hCG defined as pregnancy index), compared and were analyzed with chi- square test. The background variables between study group and control group also were compared by t-student test and chi-square test.

## Results

The studied patients had no history of abortion or pregnancy. Mean age were 29.5 and 30.8 years in the study and control group respectively (p>0.05) with the range of 20-40 years. The mean duration of infertility in the study group were 8.3 and in the control group 7.5 years. The main causes of infertility were as follow: tubal factors 31.5%, male factors 45%, ovulatory factors 18.5% and unexplained factors 15%.

The minimum and maximum retrieved oocytes from patients were 3 and 12. The numbers of transferred embryo were between 2 and 4. This study indicated embryo implantation followed by positive β-HCG test in 7 (14%) patients in ritodrine group and 8 (16%) patients in control group (p>0.05). There was not a significant difference in ritodrine group in comparison with control group. One patient in ritodrine group had minor side effects such as head ache and vertigo. 

## Discussion

As the results of this study indicate, ritodrine has not significant effect on implantation rate in IVF-ET cycles. Many chemicals were investigated for increase implantation rate and clinical pregnancy in IVF-ET (12, 13). 

Gholami and colleagues (5) compared the pregnancy outcome in patients undergoing IVF-ET cycles, using human derived follicle-stimulating hormone (FSH) or recombinant FSH for ovarian stimulation protocols. They results did not demonstrate a difference between the use of h-FSH vs r-FSH for ovarian stimulation in terms of pregnancy outcome, in good prognosis patients undergoing their first IVF-ET procedure. Moon and colleagues reported piroxicom increased the implantation rate and clinical pregnancy in the patients candidate for IVF-ET.

However, Dal Prato and Borini (10) indicated that, administration of a single dose of piroxicam before embryo transfer has no additional effect on pregnancy outcome in patients receiving adequate doses of progesterone for luteal phase supplementation after IVF or ICSI.

On the other hand, in a limited trial, an oxitocine antagonist (Atociban) was administered on 14^th^ day of endometrial synchronization for oocyste donation. The treatment decreased the uterine contractile activity and resulted in successful embryo implantation and a normal twin diamniotic pregnancy (7). It was concluded that Atosiban may improve uterine receptivity during ET and may increase success rates of advanced infertility treatment procedures, but this report was only in one case and could not be generalizes to all patients. Gorkemli and colleagues in their study in 2004 concluded that adding estradiol to progesterone in luteal phase may increase implantation rate in IVF-ET cycles (12).

Amanda and Garas, in 2006 reported that neither implantation rate nor pregnancy rate increased with paracetamol and diclofenac (13). In 2003 Peinheiro and colleagues (14) found that beta-2-adrenergic will not increase the implantation rate in ICSI cycles. They applied both ritodrine and terbutaline in divided groups. The result of their study is in agreement with our study. As we see different studies were done to identify best medication for improving ART outcome, but a definitive answer has not found yet.

NSAIDs such as paracetamol, indomethacin, and diclofenacs, and beta agonists such as ritodrine and terbutaline are the most examined drugs and their positive effect is not proven definitely yet (10, 13, 15) . Some reports indicated controversial results on the effect of peritalsis of uterus and its contractibility on the rate of implantation. Some studies report anticholinergic agents significantly suppress sporadic myometrial contractions and uterine peristalsis (6) and even adequate uterine contractility may provide for gamete/embryo transportation through the utero-tubal cavities and successful embryo implantation in spontaneous or assisted reproduction. Inadequate uterine contractility may lead to ectopic pregnancies, miscarriages, retrograde bleeding with dysmenorrhea and endometriosis (16). Therefore, the questions about uterine contractions control have some roles on the embryo implantation, remained open for more studies. 

## Conclusion

This study indicated that, ritodrine has not significant effect on the implantation rate in the IVF-ET technique. We recommend extended researches to find out a certain medication for increasing ART outcomes.

## Note

***Registretion ID in IRCT: IRCT201105316665N1***
